# Factors determining ultra-short-term survival and the commencement of active treatment in high-grade serous ovarian cancer: a case comparison study

**DOI:** 10.1186/s12885-021-08019-9

**Published:** 2021-04-08

**Authors:** Amy Hawarden, Bryn Russell, Mary Ellen Gee, Fatima Skayali, Andrew Clamp, Emma Jayne Crosbie, Richard John Edmondson

**Affiliations:** 1 Division of Cancer Sciences, Faculty of Biology, Medicine and Health, Manchester Academic Health Science Centre, University of Manchester, St Mary’s Hospital, Research Floor, Oxford Road, Manchester, M13 9WL UK; 2 Department of Obstetrics and Gynaecology, Manchester Academic Health Science Centre, St Mary’s Hospital, Central Manchester NHS Foundation Trust, Manchester Academic Health Science Centre, Level 5, Research, Oxford Road, Manchester, UK; 3 Department of Medical Oncology, The Christie NHS Foundation Trust, Wilmslow Road, Manchester, M20 4BX UK

**Keywords:** Ovarian cancer, Survival

## Abstract

**Background:**

Despite improvements in median survival some patients with advanced ovarian cancer die within 100 days of diagnosis; the reasons for which remain poorly understood.

Here we investigate if ultra short-term survival can be explained by patient characteristics or treatment pathways.

**Methods:**

A nested case comparison study was used to examine differences between patients with high grade serous ovarian/fallopian tube cancer who died within 100 days (*n* = 28) compared to a comparison group of patients matched for histology and including any survival greater than 100 days (*n* = 134).

**Results:**

Cases and comparison patients had similar ages, BMI, ACE-27, deprivation indices, and distribution of disease on CT. There were no significant delays in time to diagnosis or treatment (*p* = 0.68) between the groups.

However, cases had lower serum albumin, haemoglobin and higher platelet counts than matched comparison patients (*p* < 0.0001) and a worse performance score (*P* = 0.006).

**Conclusion:**

Patients who die rapidly after a diagnosis of ovarian cancer are only slightly older and have similar pre treatment frailty compared to patients whose survival approaches the median. However they do appear to undergo greater physiological compromise as a result of their disease.

**Supplementary Information:**

The online version contains supplementary material available at 10.1186/s12885-021-08019-9.

## Background

Ovarian cancer is best regarded as a term used to describe a heterogeneous set of pathologies with mixed prognosis. Although the overall median survival for patients with ovarian cancer is approximately 44 months the range of survival is very wide [1], reflecting the possible presence of multiple sub groups within this overall population. Even within the commonest subtype, high-grade serous cancer (HGSOC), there is heterogeneity in biology and prognosis [1, 2].

Women who survive less than 2 years from diagnosis have been termed short term survivors (STS) [2]. However, there is a small but important group of women who suffer a very rapid decline, surviving less than 100 days who may be termed ultra short-term survivors (USTS). With the first line treatment of ovarian cancer combining both surgery and chemotherapy, and spanning over approximately 150 days, none of the USTS group, by definition, will complete treatment, and a proportion will never commence treatment at all. The reasons behind this early death are poorly understood, but this group are important as they could potentially benefit from novel interventions.

The National Cancer Intelligence Network in the UK identified a group of ultra short-term survivors (USTS) [3] and showed an association with age, emergency presentation and socioeconomic status, a finding confirmed by others [4, 5]. Despite a widespread belief that rapid decline and poor outcome is associated with delays to both diagnosis and subsequent treatment, there is little evidence to support this claim. Urban et al. attempted to identify predictors of very poor outcome in patients with advanced disease. Patients dying within 90 days of diagnosis tended to be older, have increased co-morbidities, present with stage IV disease, and were less likely to have accessed specialist care [4]. However, this study was not conducted within the context of a comprehensive universal care system such as the UK National Health Sevice (NHS).

We therefore hypothesised that the rapid decline and death of patients with high-grade serous ovarian cancer would be attributed to higher levels of background co-morbidities, and delays in presentation, diagnosis and treatment compared to patients who survived longer. We therefore carried out a case comparison study of patients who died within 100 days of diagnosis (Ultra short-term survivors (USTS)) matched to a comparison group made up of patients who survived longer than 100 days.

## Methods

We included all patients referred to a tertiary treatment centre multi disciplinary team (MDT) meeting with high-grade serous ovarian cancer between 2013 and 2015 inclusive, irrespective of stage, thus including all patients with ovarian cancer within our geographical catchment area. All the patients presenting to the MDT underwent thorough investigation, including imaging, following national guidance [6]. All cytological and histological samples were assessed by two independent consultant histopathologists, one being a specialist in gynaecological cancers. All histological samples were assessed macroscopically, microscopically and underwent immunohistochemistry staining for p53, WT-1, oestrogen receptor, PAX-8, CK7 and CK20. In patients where histology was not available, cytology was used to make a diagnosis (from ascites or pleural effusions) and diagnostic methods included immunocytochemistry with the panel outlined above. In cases where the patient died before any samples were obtained, the diagnosis was made on clinical grounds by consensus between consultant radiologists, gynaecological-oncologists and clinical oncologists, based upon imaging. Patients’ management was subsequently personalised to offer primary surgery, neoadjuvant chemotherapy or best supportive care as appropriate.

The set of patients who died within 100 days of diagnosis (ultra short-term survivors, USTS) was identified. The USTS patients were then matched using a 1:5 ratio with the remainder of the cohort to generate a comparison group. We intentionally only matched for histology (high-grade serous) to facilitate evaluation of all possible differences between the two groups. Detailed patient level data were then collected for all patients. All collected variables were chosen on a pragmatic basis as likely to represent those data items routinely available to a treating clinician at the time of presentation, including those that reflect premorbid background fitness and co-morbidities, as well those that reflect the impact of disease. Data items were chosen that have already shown prognostic significance in other studies of ovarian cancer.

### Patient co-morbidities and baseline clinical characteristics

We collected the patient age, body mass index (BMI), Adult comorbidity evaluation-27 index (ACE-27) and index of multiple deprivation score (IMD), at the time of presentation. These factors were chosen to represent surrogate markers of general health before onset of disease, generally defined as “individual effects”. Although BMI can be affected by the presence of ascites or cachexia, it remains a useful marker, especially for extremes of weight categories. The ACE-27 score quantifies co-morbidities present at the time of diagnosis. The score ranges from grade 0 (no comorbidities) to 3 (severe comorbidities) [7]. This score does not take into account the current acute state of the patient, but instead acts as a background marker of fitness. The IMD score provides a decile ranking of deprivation for each geographical area of 1500 residents in the UK, where 1 is the most deprived and 10 is the least deprived. The score encompasses income, employment, education, health, including access to healthcare, crime, barriers to housing and services, and living environment to give an overall marker of deprivation [8].

### Treatment received

The active treatment of high stage high-grade serous ovarian cancer follows one of two pathways, each including both surgery and chemotherapy. Primary debulking surgery (PDS) followed by six cycles of platinum based chemotherapy was recommended for patients where complete cytoreduction was considered feasible. Neoadjuvant chemotherapy (NACT) with delayed or interval debulking surgery (IDS) was recommended for patients with apparent inoperable disease at presentation.

Treatment pathways were decided, in conjunction with the patient, by a team comprising six surgical gynae oncologists and three medical oncologists. For patients treated at Saint Mary’s, 45% of patients with advanced disease receive primary surgery and the suboptimal (greater than 1 cm residual disease) cytoreduction rate for patients undergoing surgery during the study period was 7% in both the primary and the delayed primary setting.

### Disease effect

To ascertain the disease burden at the time of presentation, we recorded the FIGO stage of disease [9], tumour distribution reported on pre-treatment CT scans, patient blood parameters, and performance status (PS). These factors were chosen to represent surrogate markers of disease burden generally defined as “tumour effects”.

Performance status (PS) is a WHO recognised tool widely used as a measure of fitness for treatment in oncology patients. It is useful to assess the acute fitness of a patient, but does not take into account co-existing co-morbidities. It is graded between 0 and 5, 0 being fully active and 5 being dead [10].

The blood parameters (haemoglobin, platelet, lymphocyte, neutrophil, albumin and CA 125) were recorded for both groups at initial presentation, to avoid any bias created by clinical intervention, such as blood transfusion. Although median albumin and haemoglobin levels decrease in an aging population, [11] these effects are small and given that both groups had very similar age ranges, no adjustment for age was made. These blood parameters were also selected as they are a routine part of the established treatment pathway.

There remains a lack of consensus upon an accurate way to assess tumour volume or distribution pre-operatively. Therefore, the diagnostic CT scan reports, generated by specialist radiologists, were mined to generate a radiology score, based on presence or absence of disease in up to 30 anatomical sites, adapted from [12–14], supplementary Table 1.

### Statistics

All data were collected, retrospectively, utilising both paper and electronic notes, from both referral hospitals, and tertiary centres. All patient identifiable data were encrypted so as to maintain confidentiality.

Data were analysed in excel and Graphpad, using Mann-Whitney U, and T tests to ascertain correlation between groups.

## Results

**Fig. 1:**
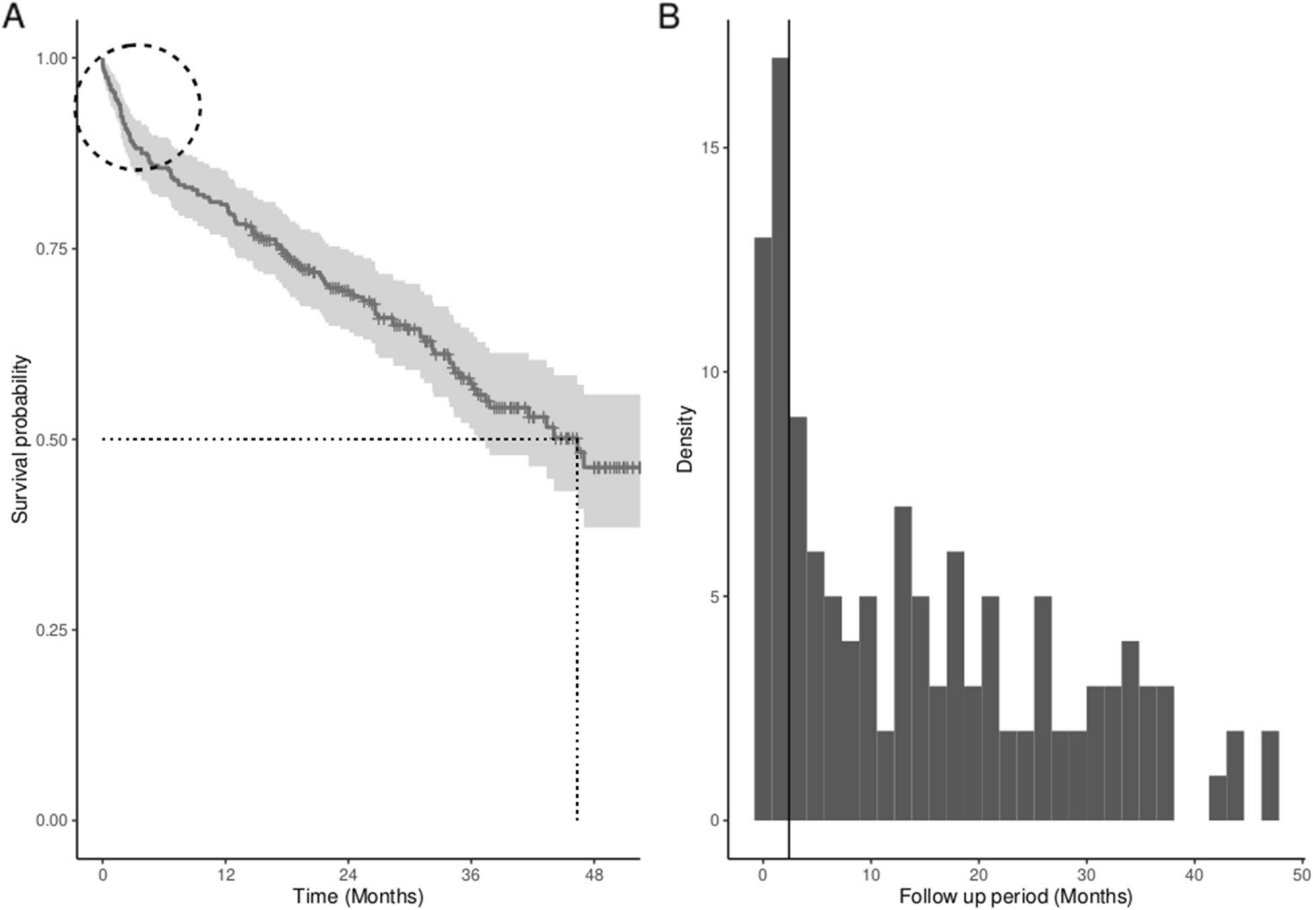
**a** Kaplan Meier survival curve for entire cohort in which the initial steep decline identifies patients with ultra short-term survival and (**b**) histogram demonstrating a clear incident peak of early deaths

**Fig. 2:**
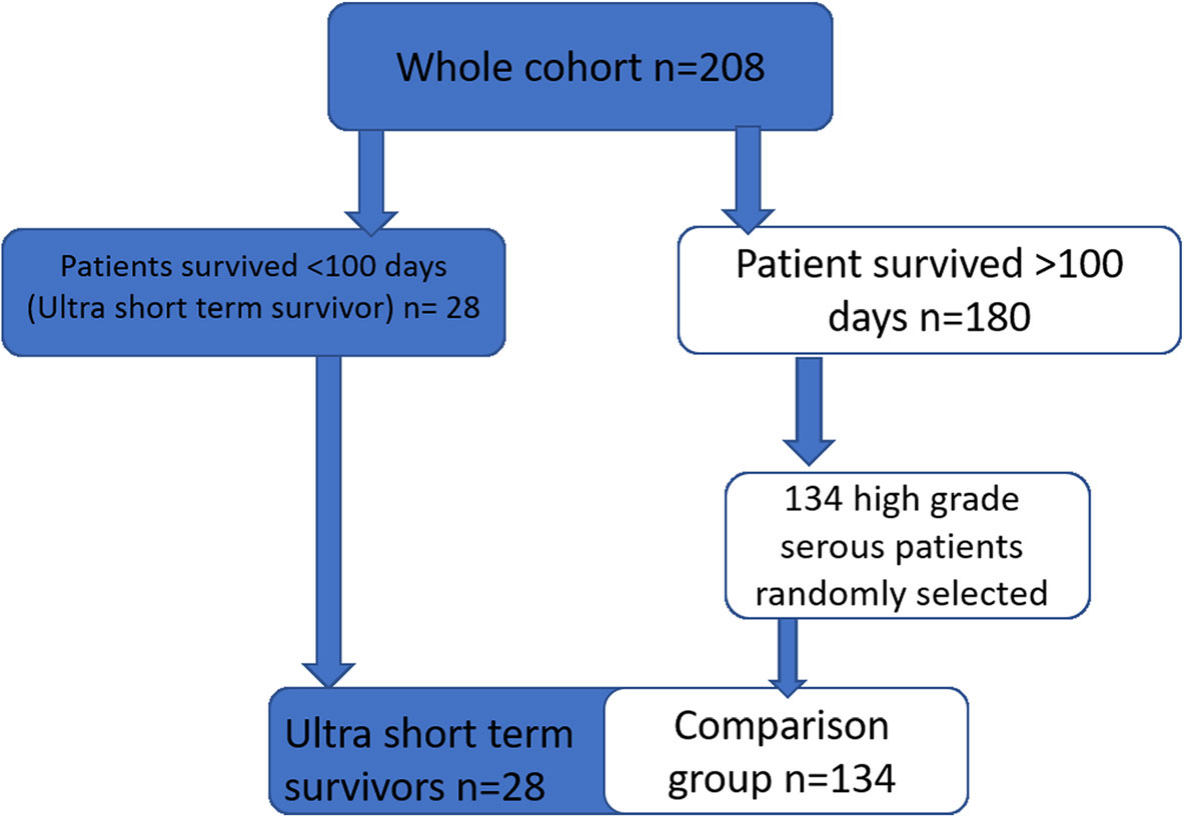
consort diagram describing the creation of our two distinct groups for comparison from our entire patient population presenting to our gynaecology MDT with HGSOC over a three year period. The 28 USTS patients represent 13.5% of the ovarian cancer population. The comparison group were matched by histological grade only (HGSOC) and were randomly selected from our entire patient population

Between 2013 and 2015 inclusive, 208 patients were diagnosed with high grade serous ovarian cancer in our tertiary unit. 28/208 (13%) died within 100 days of diagnosis (ultra short-term survivors, USTS). This group represented a discrete cohort which is demonstrated, on a Kaplan-Meier survival curve for the entire patient population, as a steep initial decline, Fig. 1a,b. One hundred thirty-four patients were then matched by histology on a 1:5 ratio, from the remainder of the dataset, to generate the comparison group, Fig. 2.

**Fig. 3:**
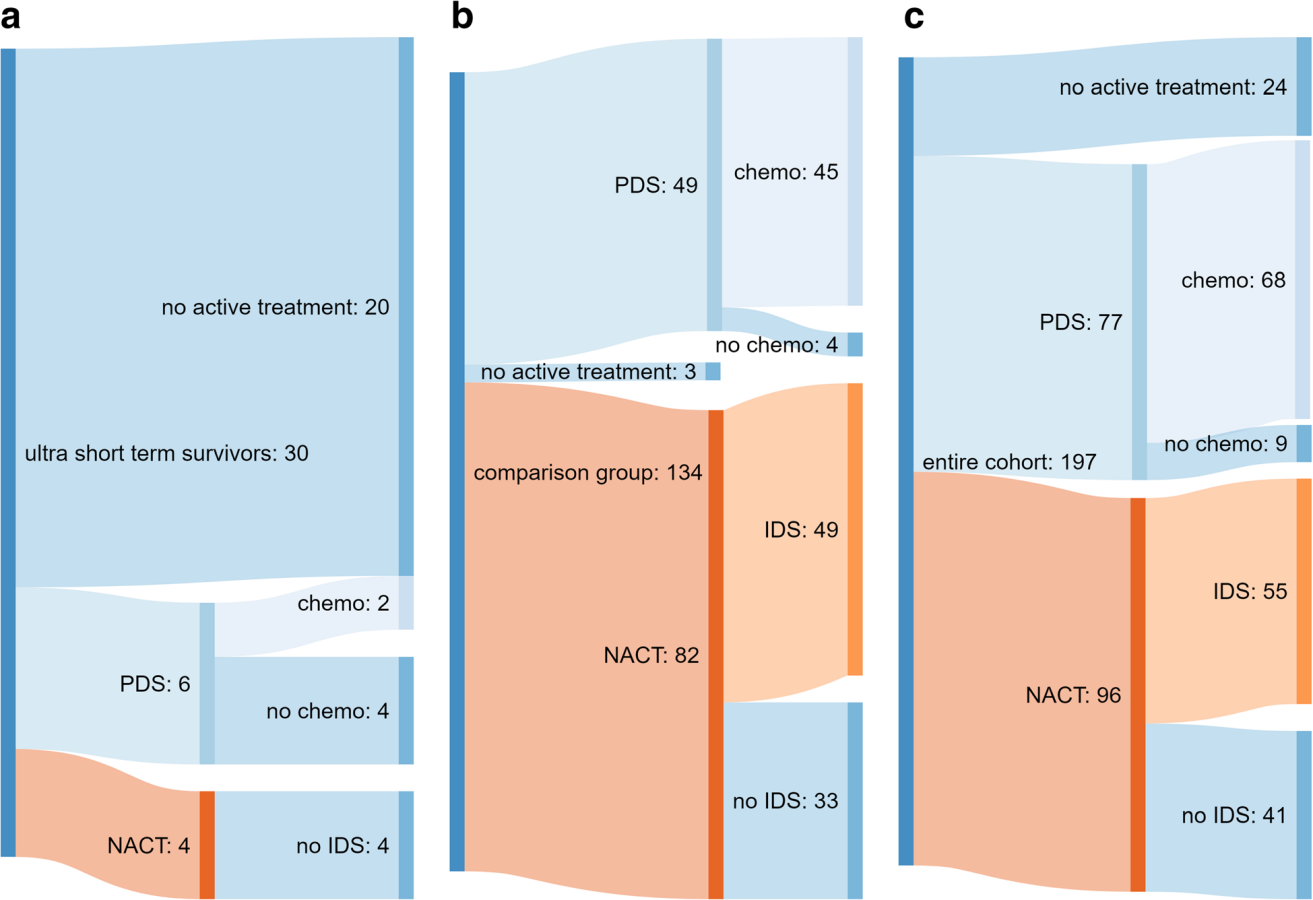
Sankey diagrams demonstrating the difference in treatment regimens received by each group, including (**a**) the entire cohort (excluding 11 of the 208 patients for whom treatment was not known). **b** USTS cases, of whom only 10/28 (36%) commenced treatment, in contrast to (**c**) comparison patients. Of whom 131/134(98%) commenced active therapy. Of those that underwent PDS, only four did not receive adjuvant chemotherapy. Of the 127 patients who underwent chemotherapy, 103 (81%) completed all 6 cycles

In the USTS group, 20/28, (71%) received no active treatment, Fig. 3b. Of the eight patients who did commence active treatment (6/28 PDS, 4/28 NACT), all died before completion of the treatment pathway. Conversely, in the comparison group, 131/134 (98%), commenced and completed first line treatment with 81% of patients completing six cycles of chemotherapy and undergoing an operation, the remainder having chemotherapy alone, Fig. 3c.

In order to ascertain the reasons between the different treatment patterns in the two groups, we then compared patient characteristics and disease burden at the time of presentation, with the aim of identifying delays within treatment pathways.

### Patient co-morbidities and baseline clinical characteristics

Although the USTS group were slightly older than the comparison group (median age 73 vs 67, *p* = 0.049) the range was similar (37–84 vs 37–90) and there were no significant differences seen between BMI (*p* = 0.083), ACE-27 score of co-morbidities (*p* = 0.34) or deprivation score (*p* = 0.27), Table 1. This suggests that the ultra short-term survivor group, although being slightly older, are not a cohort of patients who are inherently more frail. Therefore, the pre-morbid state demographic parameters have little or no effect upon survival or ability to receive treatment at the time of presentation.

**Table 1:** Pre disease characteristics of cases and controls

***r***		USTS group	Comparison group	***p*** value
**n**		28	134	
**Age (years)**	Median	73	67	0.049*
	Range	37-84	37-90	
**ACE score**	Median	1	1	0.337
	Range	0-3	0-3	
**IMD**	Median	3	4	0.2681
	Range	1-9	1-10	
**BMI**	Median	25	25	0.832
	Range	19-56	17-35	

### Patient referral pathway

The tertiary care pathway can be defined as the time taken by the gynaecological oncology specialist team to make a diagnosis and formulate a treatment decision, and includes the time taken for investigations such as radiology and undertaking biopsies. This time was calculated and compared between the two groups in order to establish whether a prolonged pathway could explain their low levels of active treatment and poor outcome.

**Fig. 4:**
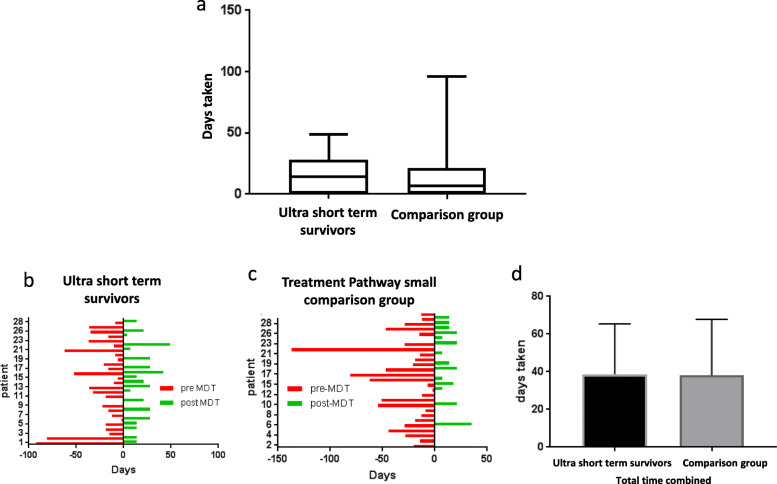
**a** comparison of time taken in the tertiary patient pathway for the two groups, demonstrating no significant difference between the time taken to reach treatment decision in tertiary care between the USTS group and the comparison group. Patient level bar charts showing time spent pre and post MDT for each patient in the (**b**) USTS group and (**c**) a randomly selected sub cohort (*n* = 28) of comparison patients. The red ‘pre-MDT’ bar represents time taken from presentation in secondary care to discussion at MDT, with the green ‘post-MDT’ bar representing time from first discussion to diagnosis/death. **d** comparison of total time taken from presentation to commencing treatment/death showing no difference between the timeline in the two groups

There were no significant differences between the two groups, with the median time taken to make a treatment decision by the tertiary gynae oncology team being 33 days in the USTS group vs 27 days in the comparison group (*p* = 0.68), Fig. 4a.

We calculated the time taken between patient’s presentation to primary care and referral to tertiary care, seen as ‘pre MDT’, and time taken from first discussion at MDT to gaining diagnosis or death, seen as ‘post-MDT’. We found no difference between the two groups for either pre-MDT (*p* = 0.54) or post-MDT (*p* = 0.14), Fig. 4b and c.

When all timelines are combined, and the total time from initial presentation in primary care to diagnosis/death, there was no difference between the two groups, Fig. 4d.

### Disease effect

Next, we compared the relationship between disease burden and survival between the two groups, Table 2. The stage of disease at diagnosis between groups showed no difference, (7% stage I-II, 93% stage III/IV), with only slightly more stage IV disease in the USTS group. However, in both groups the majority of patients presented at stage III, Table 2.

**Table 2:** Disease distribution from CT scans stratified by FIGO stage. Number of sites of disease was determined by reanalysis of CT scans

**a**				
**Parameter**		**USTS group**	**Comparison group**	**Whole cohort**
***N***		28	134	208
**Stage (FIGO)**	I - II	2 (7%)	9 (7%)	26 (13%)
	III	16 (57%)	95 (71%)	137 (66%)
	IV	10 (36%)	30 (22%)	45 (22%)
**b**				
**Stage**		**USTS group**	**Comparison group**	***p*** **value**
**I + II**	Mean no of sites	1	1	0.6
**III**	Mean no of sites	3.6	2.9	0.18
**IV**	Mean no of sites	4.3	3.7	0.35

As a surrogate marker for tumour load, we compared sites of disease as assessed by CT between groups. There was no difference in disease distribution between the groups, either by site of disease, or number of sites involved, even when stratified by stage, suggesting the USTS group did not have a larger disease burden at the time of presentation, Table 3.

**Table 3:** Shows comparison of disease effect characteristics between the ultra short-term survival group and control patients

Parameter		Ultra short term survivors	Comparison group	***p*** value
**Performance score**	Median	2	1	0.006**
	Range	0-4	0-3	
**Hb**	Mean	115	118	0.1538
	Range	92-152	81-148	
	SD	15	18	
**Lymphocytes**	Mean	1.0	1.79	<0.0001 ****
	Range	0.37-1.16	0.36-3.67	
	SD	0	0.76	
**Neutrophils**	Mean	10	4.87	<0.0001 ****
	Range	4.8-19.3	0.55-20.52	
	SD	4	2.6	
**Albumin**	Mean	28	36	<0.0001 ****
	Range	11-43	19-45	
	SD	8	4.6	
**Plts**	Mean	533	313	<0.0001 ****
	Range	192-1145	81-714	
	SD	236	121	
**CA 125**	Mean	2714	939	0.0223*
	Range	53-30865	10-12622	
	SD	5880	1710	

Finally, we assessed the impact of the disease upon patient physiology and function using a series of surrogate markers. The USTS group had poorer performance status compared to the comparison group (*p* = 0.006). Patients with a worse performance status were less likely to receive surgical management (21% in USTS vs 73% in comparison group), however some patients with low performance scores did not receive surgery, and some with high performance scores did receive surgery, suggesting that PS is not the only confounding factor in surgical fitness.

There were also significant differences in blood parameters between the groups, with differences in platelet count, albumin, lymphocyte and neutrophil levels being significant (*p* < 0.0001), Table 2. These differences remained significant when stratified for stage, supplementary Table 1.

Finally, to ensure that these analyses described above remained valid for all patients, including long term survivors the control group was divided into patients with short term survival (STS – 100 days to 2 years) and long term survivors (LTS – greater than 2 years). This showed an equivalent number of patients in each group (*n* = 66 and *n* = 68 respectively). Small but statistical differences between the two groups were limited to performance status, pretreatment lymphocyte count and pretreatment serum albumin levels, suggesting that the differences seen between USTS survivors and controls applies to both STS and LTS groups equally (supplementary Table 2).

## Discussion

We identified a cohort of patients, comprising 13.5% of our patients with high-grade serous ovarian cancer, who die within 100 days of diagnosis, with most not receiving any form of active treatment, and none completing treatment. Here we attempted, for the first time, to identify if there were significant clinical differences between those patients who suffered early demise and did not commence treatment, compared to a comparison group within the context of a universal health care system in which all patients were managed within a single clinical pathway.

Although patients in the USTS group were on average slightly older, there were no significant differences between their co-morbidities suggesting that this is not a group that is inherently frail or unwell prior to the onset of disease.

Socioeconomic status did not significantly differ between our cohorts. Although socio-economic status has been associated with overall survival [4, 5, 15] our data suggest that this is not an important factor in determining short term outcome and the commencement of treatment, at least in the context of a universal health care system.

Contrary to our hypothesis that the USTS group would represent a cohort in whom treatment had been delayed in some way, or who presented late, no significant delays were identified within this group. Although it is well recognised that standards of care and adherence to clinical guidelines are important in determining outcome [16], our study was a single centre study in which all patients were managed by a single clinical pathway. Therefore the USTS group received less treatment, and did especially poorly, despite their care being determined using identical treatment protocols.

At the time of presentation, the distribution of disease does not appear to differ between the two groups, thus refuting the argument that the USTS group fare poorly due to delayed presentation and therefore more widespread disease and a higher disease burden. In both groups the majority of patients present at stage III, and have similar disease distributions, when stratified by stage. Despite presenting with similar disease distribution, the USTS are much less likely to receive or complete treatment.

However, we did show significant differences between performance status and diagnostic blood parameters. These parameters are markers of the patient’s biological and physical response to tumour and are unlikely to be explained by other factors such as bone metastasis which occurs in less than 0.5% of patients with high-grade serous ovarian cancer [17]. This suggests a differing physiological response to disease between the cohorts that cannot be explained by disease distribution or FIGO stage. We propose that the differences in the cohorts may be determined by differences in tumour biology. Given that this cohort has never been studied in any depth on a biological level, it is possible there are unique and thus far unidentified, genomic or other molecular differences between these tumours. These differences would be best studied via prospective tissue collection studies.

In this study, given the paucity of tissue samples available from this cohort we were unable to study the biology of the tumours within the USTS group. We were not even able to comment upon germline BRCA status as none of the USTS group underwent germline testing, likely due to insufficient time for genetics referral before death. These observations highlight the challenges of studying this group of patients.

Until now identifying these patients to allow recruitment into studies has not been possible and obtaining histological biopsy for diagnosis that yields sufficient material for translational studies is difficult. Little is known regarding the chemotherapy responsiveness of these tumours as most previous work has been enriched for patients with a much better prognosis and it is thus important that translational work is focussed on tumours from patients at high risk of early demise who may then be able to be included in future clinical trials of first line therapy and new targeted therapies.

The landscape of ovarian cancer has been refined over recent years with data from the TCGA and other consortia outlining classifications based on DNA damage repair status [18, 19], mutation profiling [18], gene expression [20], and copy number changes [21]. Taken together it is recognised that poor prognosis groups are associated with tumours with homologous recombination competency, cyclin E amplification, and specific copy number signatures. The stromal matrix is also linked with disease progression in ovarian cancers [22]. What remains unclear however, is whether patients with USTS have tumours which have a preponderance of these already established poor prognosis features or whether they represent a group of tumours with an, as yet, undescribed biology.

It is hoped that future studies answering these questions will allow targeted clinical trials to facilitate tailoring of treatment for the patients who, at present, have the worst prognosis.

## Conclusions

A small but significant proportion of patients die rapidly after a diagnosis of ovarian cancer. Here we have shown that these patients are neither older nor inherently more frail than patients whose survival approaches the median. However they do appear to have disease which causes greater physiological changes.

## Supplementary Information

**Additional file 1: Supplementary Table 1**: Disease effect characteristics, stratified by stage. **Supplementary Table 2**: Comparison of short term and long term survivors within control group.

## Data Availability

There are no publicly available data from this study.

## Abbreviations

ACE27: Adult comorbidity evaluation-27 index
BMI: Body mass index
BRCA: Breast cancer gene
CT: Computerised tomography
FIGO: Federation international gynecology & obstetrics
HGSOC: High-grade serous ovarian cancer
IDS: Interval debulking surgery
IMD: Index of multiple deprivation
MDT: Multidisciplinary team
NACT: Neo adjuvant chemotherapy
NHS: National health service
PDS: Primary debulking surgery
PS: Performance status
STS: Short term survival
TCGA: The cancer genome atlas
UK: United Kingdom
USTS: Ultra-short term survival
WHO: World health Organisation
