# Correction to: Factors determining ultra-short-term survival and the commencement of active treatment in high-grade serous ovarian cancer: a case comparison study

**DOI:** 10.1186/s12885-021-08369-4

**Published:** 2021-05-26

**Authors:** Amy Hawarden, Bryn Russell, Mary Ellen Gee, Fatima Kayali, Andrew Clamp, Emma Jayne Crosbie, Richard John Edmondson

**Affiliations:** 1 Division of Cancer Sciences, Faculty of Biology, Medicine and Health, Manchester Academic Health Science Centre, University of Manchester, St Mary’s Hospital, Research Floor, Oxford Road, Manchester, M13 9WL UK; 2 Department of Obstetrics and Gynaecology, Manchester Academic Health Science Centre, St Mary’s Hospital, Central Manchester NHS Foundation Trust, Manchester Academic Health Science Centre, Level 5, Research, Oxford Road, Manchester, UK; 3 Department of Medical Oncology, The Christie NHS Foundation Trust, Wilmslow Road, Manchester, M20 4BX UK

**Correction to: BMC Cancer 21, 378 (2021)**

**https://doi.org/10.1186/s12885-021-08019-9**

Following publication of the original article [1], the authors identified an error in the author name of Fatima Kayali.

The incorrect author name is: Fatima Skayali

The correct author name is: Fatima Kayali

The Contributions section has therefore also been updated as follows:

RJE and AH devised the study, AH, MEG, & FK extracted data, AH & BR analysed data, AC & EJC provided interpretation and assisted writing the manuscript. All authors have read and approved the manuscript.

The author group has been updated above and the original article [1] has been corrected.
