# Supplementary material for: Factors determining ultra-short-term survival and the commencement of active treatment in high-grade serous ovarian cancer: a case comparison study
Source: BMC Cancer. 2021 Apr 8;21:378. doi: 10.1186/s12885-021-08019-9 (PMC8034099; doi:10.1186/s12885-021-08019-9)
Supplement: Supplementary file 1 — Additional file 1: Supplementary Table 1: Disease effect characteristics, stratified by stage. Supplementary Table 2: Comparison of short term and long term survivors within control group. [file 12885_2021_8019_MOESM1_ESM.docx]

**Factors determining ultra short term survival in high grade serous ovarian cancer**

**Authors:** A Hawarden B Russell, ME Gee, F Skayali, A Clamp, G Jayson, EJ Crosbie, RJ Edmondson

Supplementary Data

Supplementary table 1: Disease effect characteristics, stratified by stage


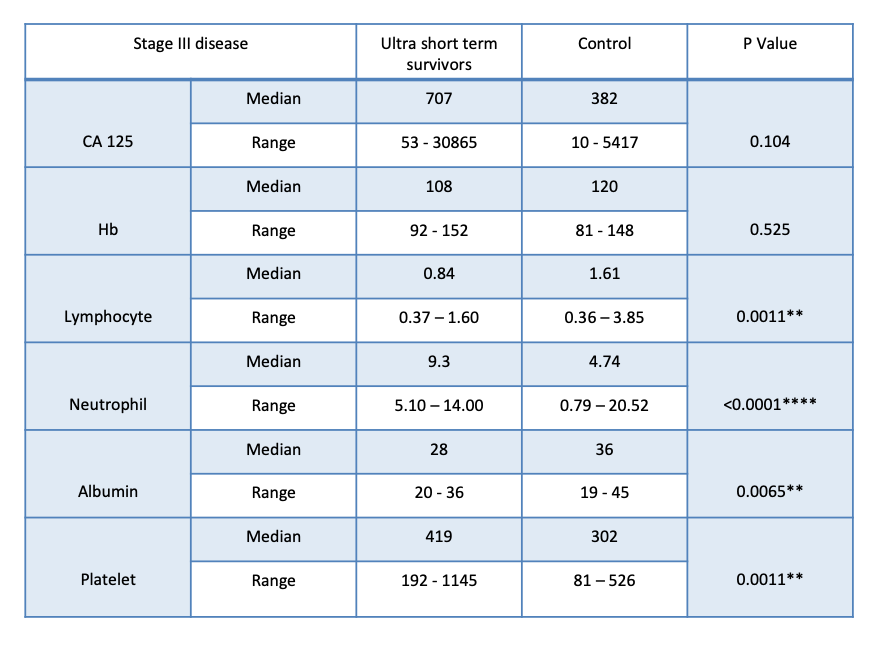


Supplementary table 2: Comparison of short term and long term survivors within control group

|  | **USTS^1^ (n=28)** | **STS^2^ (n=66)** | **LTS^3^ n=(68)** | **p^4^** |
| --- | --- | --- | --- | --- |
| Age (years) | 73 (37-84) | 69 (41-90) | 66 (37-86) | 0.3129 |
| Stage I+II | 2 (7%) | 2 (3%) | 7 (10%) | 0.13 |
| Stage III | 16 (57%) | 46 (70%) | 49 (72%) |  |
| Stage IV | 10 (71%) | 18 (27%) | 12 (18%) |  |
| BMI | 25 (19-56) | 24 (18-35) | 26 (17-35) | 0.1075 |
| PS (0-5) | 2 (0-4) | 2 (0-3) | 1 (0-3) | **0.0139** |
| IMD (1-10) | 4 (1-9) | 3 (1-10) | 4 (0-10) | 0.3123 |
| ACE-27 (0-4) | 1 (0-3) | 1 (0-3) | 1 (0-2) | 0.1845 |
| Hb (g/L) | 118 (81-148) | 119 (81-142) | 122 (94-148) | 0.1744 |
| Plt (x10_9_/L) | 533 (192-1145) | 314 (92-714) | 293 (81-486) | 0.6009 |
| Lymph (10_9_/L) | 1.0 (0.37-1.16) | 1.51 (0.36-3.03) | 1.90 (0.52-3.67) | **0.0027** |
| Neut (10_9_/L) | 1.0 (4.8-19.3) | 4.09 (0.62-20.52) | 4.80 (0.55-11.36) | 0.1559 |
| Alb (g/L) | 28 (11-43) | 35 (19-44) | 38 (26-45) | **0.0034** |
| CA 125 (KU/L) | 2,714 (53-30,865) | 616 (20-12,622) | 199 (10-3,566) | 0.0589 |

1 USTS – ultra short term survival (less than 100 days)

2 STS – short term survival (greater than 100 days but less than 2 years)

3 LTS - long term survival (greater than 2 years)

4 all comparisons made between STS and LTS. For comparisons including USTS see tables 1 – 3. All comparisons Kruskal Wallis except stage, Chi squared
